# Editorial: Microbial Production of Biopolyesters and Their Building Blocks: Opportunities and Challenges

**DOI:** 10.3389/fbioe.2021.777265

**Published:** 2021-12-08

**Authors:** Huibin Zou, Seiichi Taguchi, David Bernard Levin

**Affiliations:** ^1^ College of Chemical Engineering, Qingdao University of Science and Technology, Qingdao, China; ^2^ CAS Key Laboratory of Bio-based Materials, Qingdao Institute of Bioenergy and Bioprocess Technology, Chinese Academy of Sciences, Qingdao, China; ^3^ Faculty of Life Sciences and Agriculture, Tokyo University of Agriculture, Tokyo, Japan; ^4^ Department of Biosystems Engineering, Faculty of Agricultural and Food Sciences, University of Manitoba, Winnipeg, MB, Canada

**Keywords:** biopolyesters, microbial biotechnology, poly(lactic acid), polyhydroxyalkanoates, biomonomers

Although polyhydroxyalkanoates were discovered over 100 hundred years ago, and have been used for many applications (Choi et al., 2020), the majority of global polyester supply still relies on traditional fossil-feedstocks, with well-developed chemical techniques and significant advantages in economy and scale. The barriers for the microbial production of biopolyesters major include: 1) high production and downstream purification costs (Wang et al., 2019); 2) limited production capacity and supply of non-food bio-feedstocks; and 3) limited diversity of monomers and final products to adapt to marketing requirements (Zheng et al., 2020).

To solve these bottleneck barriers, large members of research institutes and industries have contributed their endeavors in this developing field. Other than polyhydroxyalkanoates, semi-synthesized polylactic acid (PLA) has become another bulk commercialized biopolyester with acceptable unit cost and promising material properties (Castro-Aguirre et al., 2016). Other novel biopolyesters are also in fast development. For example, “unnatural” lactate containing polyesters can be microbially produced after systematic engineering of key enzymes and chassis strains (Taguchi et al., 2008; Choi et al., 2016). After that, new 2-hydroxy monomeric constitutes such as glycolate and 2-hydroxybutyrate could be incorporated into the polymeric backbone (Taguchi and Matsumoto, 2020). Furthermore, novel aromatic biopolyesters like d-phenyl lactate containing polyesters can also be produced by engineered strains (Yang et al., 2018).

Moreover, the diversity of bio-monomers has increased in recent years (Taguchi and Matsumoto, 2020). In addition to well-known bio-organic acids (lactic acid and succinic acid), bio-diols like 1,4-butanediol and 1,3-propanediol, have achieved high-titer production using engineered strains (Yim et al., 2011; Ju et al., 2020). These bio-monomers can be utilized as drop-in chemicals in the production of commercial polyesters like poly(butylene succinate) (PBS), poly(butylene terephthalate) (PBT), and poly(propylene terephthalate) (PPT).

In this Research Topic, a number of experts contributed their updated research outcomes or opinions regarding the strategies to improve the microbial production of polyester. From the perspective of microbial production of novel lactate containing polyesters and oligomers, Nduko and Taguchi have provided insights into the history for the development of lactate containing polyesters/oligomers, and the applications of variable lactate containing polyesters/oligomers (as macromonomer building blocks). Relating to the use of non-food feedstocks in the bioproduction of polyesters, Sun et al. and Moriya et al. present their updated studies on the microbial production of poly(3-hydroxybutyrate) from a broader-range of non-food substrates. Relating to the microbial production of medium chain-length polyhydroxyalkanoates, Scheel et al. reported a fermentation protocol which can be applied to control the copolymer composition to exhibit increased flexibility and elasticity in a series of medium chain-length poly(3-hydroxyalkanoates). Harada et al. present a study on the microbial production of medium chain-length poly(3-hydroxyalkanoates) by application of an engineered polyhydroxyalkanoate synthase, which significantly improved the 3-hydroxyhexanoate (3HHx) fraction in the copolymers. Dartiailh et al. studied the effects of monomer composition on the thermal and mechanical properties of medium chain-length polyhydroxyalkanoate synthesized by *Pseudomonas putida* cultured with different substrates.

It is hard to forecast the future of polyester industries, but we believe biopolyesters will have a promising future, based on: the availability of renewable and sustainable bio-feedstocks to support the bioproduction of polyesters; consumer demand for biodegradable and bioassimilatable materials based on renewable biopolymers; industries and research institutes that are eager to develop renewable and degradable polyester products; and the increasing application of advanced biotechnologies (like synthetic biology with the goal of making novel bio-monomers) and materials science techniques (like the research on biomaterials made from functional biopolyesters) that are being applied in this field.

Enjoy reading!
